# Plasmonic and Hybrid Whispering Gallery Mode–Based Biosensors: Literature Review

**DOI:** 10.2196/17781

**Published:** 2021-04-12

**Authors:** Maurizio Manzo, Omar Cavazos, Zhenhua Huang, Liping Cai

**Affiliations:** 1 Photonics Micro-Devices Fabrication Lab Department of Mechanical Engineering University of North Texas Denton, TX United States; 2 Department of Mechanical Engineering, University of North Texas Denton, TX United States

**Keywords:** plasmonic, whispering gallery mode, microlasers, biomedical, sensors

## Abstract

**Background:**

The term “plasmonic” describes the relationship between electromagnetic fields and metallic nanostructures. Plasmon-based sensors have been used innovatively to accomplish different biomedical tasks, including detection of cancer. Plasmonic sensors also have been used in biochip applications and biosensors and have the potential to be implemented as implantable point-of-care devices. Many devices and methods discussed in the literature are based on surface plasmon resonance (SPR) and localized SPR (LSPR). However, the mathematical background can be overwhelming for researchers at times.

**Objective:**

This review article discusses the theory of SPR, simplifying the underlying physics and bypassing many equations of SPR and LSPR. Moreover, we introduce and discuss the hybrid whispering gallery mode (WGM) sensing theory and its applications.

**Methods:**

A literature search in ScienceDirect was performed using keywords such as “surface plasmon resonance,” “localized plasmon resonance,” and “whispering gallery mode/plasmonic.” The search results retrieved many articles, among which we selected only those that presented a simple explanation of the SPR phenomena with prominent biomedical examples.

**Results:**

SPR, LSPR, tilted fiber Bragg grating, and hybrid WGM phenomena were explained and examples on biosensing applications were provided.

**Conclusions:**

This minireview presents an overview of biosensor applications in the field of biomedicine and is intended for researchers interested in starting to work in this field. The review presents the fundamental notions of plasmonic sensors and hybrid WGM sensors, thereby allowing one to get familiar with the terminology and underlying complex formulations of linear and nonlinear optics.

## Introduction

The term “plasmonic” describes the relationship between electromagnetic fields and metallic nanostructures [1]. Plasmonic sensors have attracted great interest from researchers and engineers alike. Surface plasmon resonances (SPRs) are electromagnetic waves that are produced when a metal nanostructure (ie, spherical or cylindrical) interacts with a dielectric material [2,3]. The interesting optical characteristics of surface plasmons have made many important contributions to the field of medicine [4]. For example, highly sensitive plasmonic sensors have been developed to detect many kinds of cancers [5], and based on the SPR concept, a plasmonic interferometer array–based sensor was developed for detecting cancers [6].

This paper discusses the physical principles in brief and introduces several methods employing plasmonic systems such as SPR and localized plasmon resonance. SPR methods have attracted great interest in biomedical applications. This technique entails observing small changes in the refractive index of the combination of dielectric materials and metal [7]. In addition, plasmonic nanoparticles and nanostructures have been used in biosensing applications. These structures are typically made of noble metals such as gold and silver [8,9]. The cytotoxicity of these metals based on their concentrations are under investigation and studies have shown potential biomedical applications for these metals at certain concentrations [10,11]. These nanostructures are used in photoacoustic imaging and phototherapy. For example, gold nanorods with varied light absorption peaks have been used in imaging and theranostics [12]. Plasmonic sensors also have been used in biochip applications and biosensors [13-15]. Other techniques such as localized plasmon resonance have also been utilized in various biomedical applications [16]. In summary, plasmon-based sensing methods are indispensable tools for sensing in the field of biomedicine. Moreover, these devices have the potential to be implemented as implantable point-of-care devices [17-19].

## Methods

We performed a literature search on ScienceDirect for studies on plasmonic and hybrid whispering gallery mode (WGM) sensors and retrieved more than 3400 articles (both research and review articles) published in the field of medicine and dentistry. According to their characteristics, sensors were grouped into 4 categories, namely, SPR, localized SPR (LSPR), tilted fiber Bragg grating (TFBG), and hybrid WGM sensors.

The search for articles related to SPR and LSPR was straightforward. The search results retrieved many articles, among which we selected only those that presented a simple explanation of the aforesaid phenomenon with prominent biomedical examples. Within these 2 fields, a third field was categorized (TFBG) due to the prominent presence of biosensor-based devices that utilize the TFBG principle.

The keyword “whispering gallery mode” was associated with the term “plasmon” and only retrieved 3 papers in the pharmacology, toxicology, and pharmaceutical fields. Therefore, for the latest category (ie, hybrid WGM sensors), the search was expanded to the engineering field and eventually 35 reports were identified. More papers were found in other fields such as physics, astronomy, material science, and chemistry.

## Results

Many papers, for example [20-33], describe the physical principle of SPR, and provide the definitions and discuss exemplary applications to illustrate how changing the refractive index can be used for sensing through the plasmonic effect and how the light is generally coupled to the biosensor. A total of 6 papers [23-27,29] illustrated that optical fibers can be used in conjunction with SPR for sensing applications. Also, 13 papers were selected to discuss SPR-based metal nanostructures [2,20-22,34-42].

LSPR is discussed based on 11 papers [36-39,43-49]. As before, the physical principle and 2 representative examples are provided to understand the main differences between LSPR and the previous methods.

Although TFBG could be associated with the SPR-based optical fiber sensing method, many different papers have been found on this topic, and therefore, a separate category (ie, tilted fiber Bragg grating) was created. Several papers are used to illustrate the physical principle and applications.

Lastly, 20 papers were used to introduce the WGM and the hybrid WGM sensing [34,35,40-42,50-63]. This type of sensing methodology was not directly related to the medical literature, but biomedical applications were proposed and the future implementation of this method is likely to become the gold standard in some areas. This review paper presents and discusses the sensing techniques, including SPR, LSPR, TFBG, and hybrid WGM, as well as their applications using representative examples in the biomedical fields.

## Discussion

### Surface Plasmon Resonance

**Figure 1:**
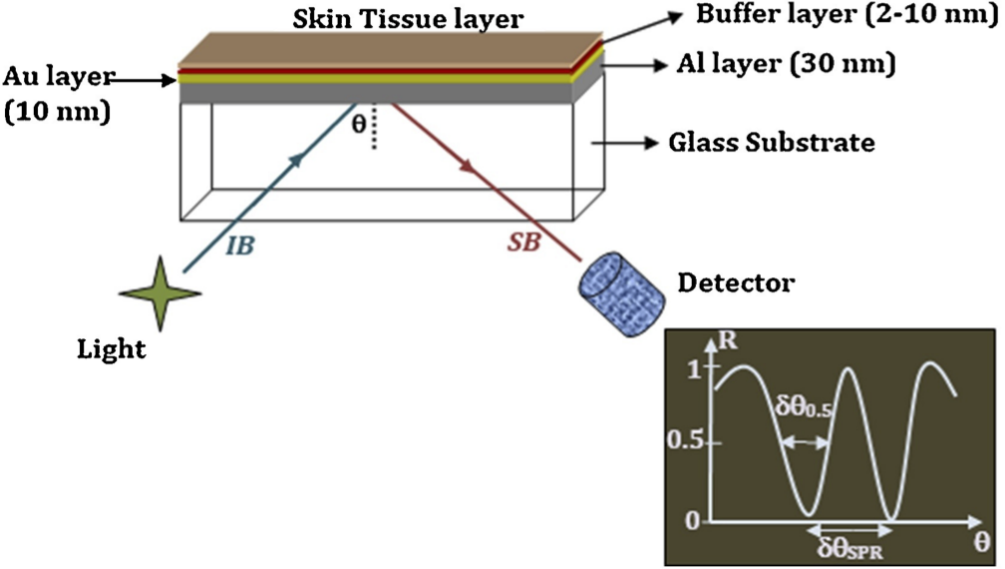
Proposed SPR sensor probe setup for the determination of refractive index of human skin tissues. IB: incoming light beam; SB: sensed light beam. Reproduced, with permission, from Elsevier [20].

The term “plasmonic” describes the relationship between metallic structures and dielectrics in an electric field. The oscillations of electrons between a metal sample and a dielectric field are referred to as SPR. The attenuated total reflection (ATR) configuration is one of the prism coupling–based SPR methods [20-22]. In ATR, a metal sheet is placed on top of a light coupling substrate, such as glass (Figure 1). The light source is then directed into a prism and a detector gathers the resonances. Therefore, the resonance is displaced as a sharp dip in the output spectrum due to the absorption of the surface plasmon wave [20]. In a previous study [20], the ATR configuration was used to monitor the refractive index of the human skin as shown in Figure 1. Besides, this same configuration has been used for monitoring humidity, where the effect of the temperature on the sensor was analyzed. The sensor consisted of a chalcogenide substrate layer, gold layer, and buffer layer [22].

Optical fiber sensing with SPR has been used to detect different kinds of biological targets such as antibodies. The phenomenon of SPR occurs on the surface of the optical fiber [23]. Optical fiber SPR methods have advantages over traditional prism methods such as the ATR technique, which can be explained as follows: optical sensors use remote sensing and optical fibers have a reasonably lower cost and a more compact size. In addition, these types of sensors provide label-free sensing with high sensitivity [24]. In some cases, finite-element methods were used to analyze the design of the optical fiber sensors [25,26].

Another class of optical fiber based on SPR uses photonic crystal fibers, which have been used as alternatives to traditional optical fibers. A notable advantage of these fibers is that they have more controllable birefringence and therefore a better control on light propagation and confinement directions [27]. Photonic crystals are dielectric materials that have a periodicity (repeated optical structure) in two or three dimensions. They are usually fabricated by etching, which can form a photonic bandgap, allowing to configure the light for different uses. The bandgap depends on the structural content of the crystal, such as refractive index and periodicity. The peak frequency shown in the transmission spectrum depends on the shape and size of the lattice defects [28].

Another method for SPR is the use of metallic nanostructures. Gold and silver nanostructures have been extensively used in past years, because they can be characterized by their size and shape [29]. Silver nanorods are suitable for biomolecular detection [30]. In addition, the iron oxide–gold nanoparticles can experience both plasmonic and magnetic phenomena, which allows for their use in different biomedical applications. One advantage of this type of particle is that it can be moved due to its magnetic property and still demonstrate plasmonic behavior. This can tremendously facilitate the analysis of biological targets [31]. In another study, a piece of portable SPR instrument was developed using nanoparticles, which was able to detect testosterone [32]. Another type of SPR sensor is the plasmonic waveguide. Plasmonic waveguide designs tend to be suitable for chipping applications. This is mainly because of their compactness and the use of SPRs [33].

### Localized Surface Plasmon Resonance

**Figure 2:**
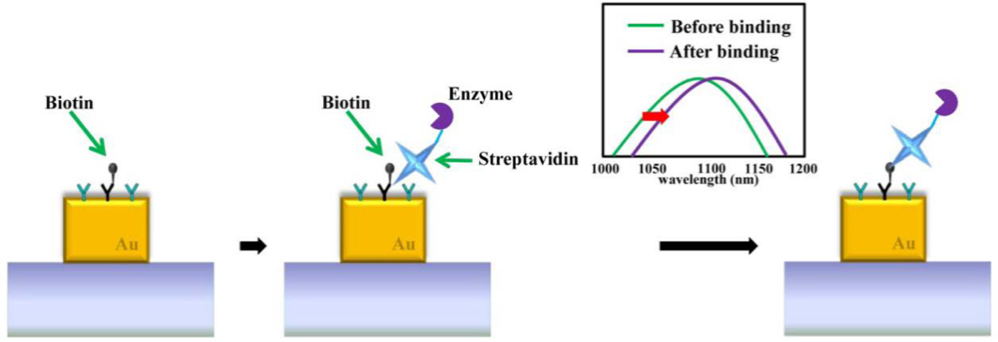
Detection of protein-protein binding event on the gold nanostructure through LSPR peak shift. Licensed under Creative Commons Attribution 4.0 by the authors [43]. LSPR: localized surface plasmon resonance.

Localized SPR (LSPR) is the amplitude of oscillation of free electrons that occurs at a certain frequency, which can be used to detect biomolecules such as proteins in real time. In one study, a gold nanoplasmonic sensor was used to detect cancer markers in clinical samples. The sensor could also detect proteins such as biotin (Figure 2). Besides, it has the potential to detect DNA [43]. LSPR is mainly related to nanostructure/nanoparticles such as nanorods. In addition, LSPR does not require coupling, for example, with prims and is easy to operate. Therefore, this method is widely used in the scientific field [44]. The type of nanostructure used has an impact on the strength of LSPR. For example, nanostars can be used for tuning the sensors and promoting a strong LSPR signal. However, structures such as nanorods and nanospheres have widely been investigated for various imaging applications [45].

**Figure 3:**
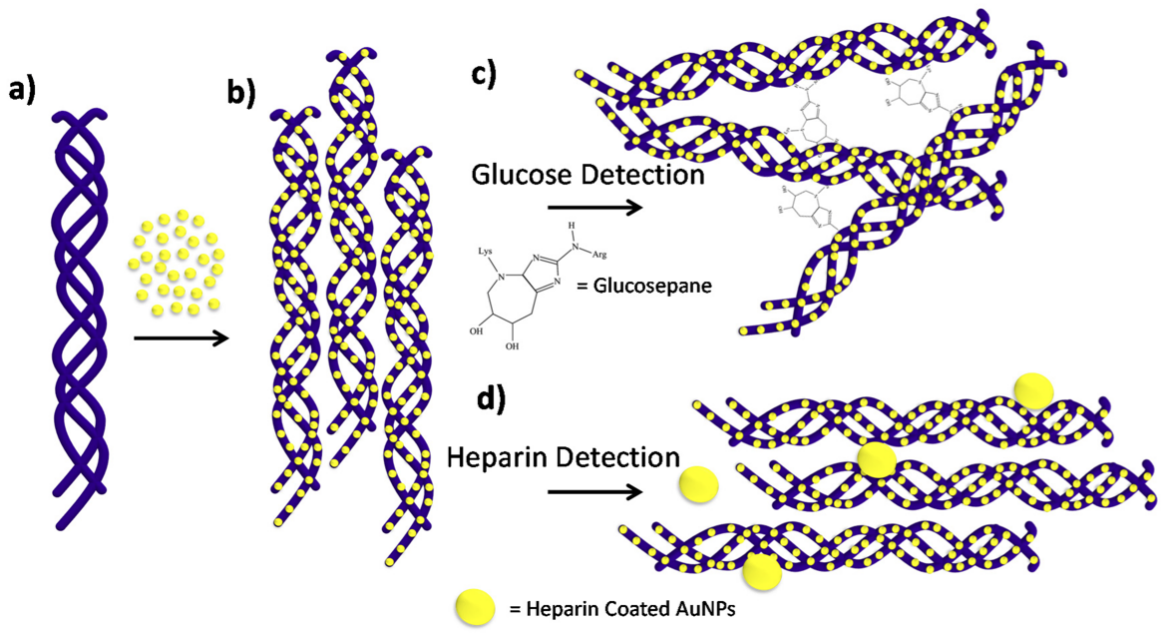
The 2 sensing schemes addressed in this work using collagen-nanoparticle conjugates. (A) The native collagen is added before the gold nanoparticles (AuNPs); (B) the collagen after it has been coated in AuNPs forming a collagen-nanoparticle scaffold; (C) in order to carry out biosensing measurements of glucose, the collagen nanoparticle scaffold is crosslinked by glucose after it has been incubated at 35°C and the covalent product glucosepane has formed; (D) lastly, the binding interactions between the collagen-nanoparticle scaffold and the heparin-coated 80-nm gold nanoparticles are used to detect heparin. Licensed under Creative Commons Attribution 4.0 by the authors [48].

Gold nanorods have been used as plasmonic sensors for detecting mercury. The deposition of mercury on the nanorods was observed by monitoring the LSPR shifts using darkfield microscopy [46]. Tao et al [47] used a gold and silver alloy nanoplasmonic device to detect mercury concentrations. LSPR has also been used to develop silk plasmonic absorber sensors [36]. In this case, silk protein is used as an insulator in the insulator–metal resonator configuration. Besides, the silk plasmonic absorber sensor was applied as a glucose sensor, which demonstrated a high sensitivity of 1200 nm/RIU (refractive index unit) and high relative intensity change [36]. Metal nanoparticles have also been used for copper detection in samples, mainly because LSPR is influenced by the morphology and size of particles. Ding et al [37] used gold nanoparticles to detect specific copper ions. Besides, LSPR sensors have been integrated into optical fiber devices. Tu et al [38] used hollow gold nanocages for LSPR optical fiber sensors. The sensor had a sensitivity of around 1933 nm/RIU. Furthermore, the sensitivity can be adjusted by changing the aspect ratio of the gold nanocages. Yousuf et al [39] developed a metal–insulator–metal configuration, which consisted of an elliptical nanorod, rectangular nanoslabs, and a metallic grating. Unser et al [48] developed a selective collagen gold nanoparticle–based sensor, which works based on the plasmonic coupling of the nanoparticles and the collagen fibrils. A redshift (toward the right side of the spectrum) in the LSPR frequency indicates the detection of glucose. Overall, the conjugates were able to detect glucose and heparin (Figure 3).

### Tilted Fiber Bragg Grating

In the configuration of TFBG, the refractive index modulation planes are in a tilted position, which helps to measure very small changes. These small changes can be fully analyzed by observing the refractive index of the fiber. The tilted grating disrupts the fiber’s symmetry, which causes some core-guided lights to be coupled that allows the cladding mode resonances to be observed. These resonances are observed as a comb of sharp dips. It has been noted that these methods can greatly increase quality factors (ie, Q values of up to 10^4^) [64].

**Figure 4:**
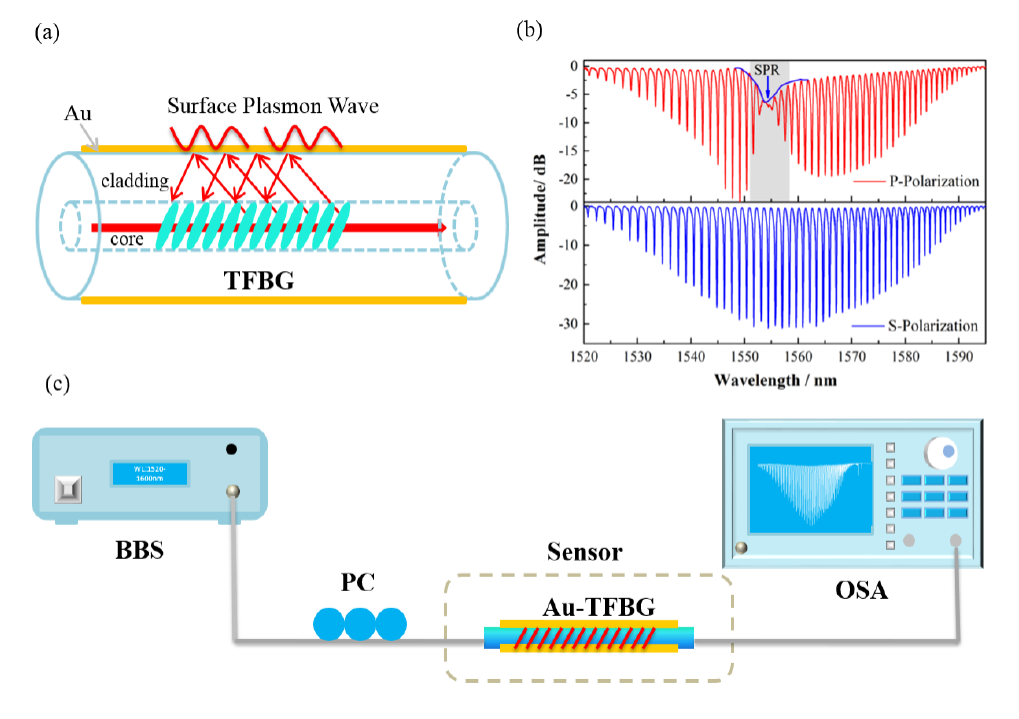
(A) Schematic of the tilted fiber Bragg grating (TFBG)-based surface plasmon resonance (SPR) (TFBG-SPR) sensor. (B) Transmission spectra of the sensor under P and S polarization. (C) Experimental setup. Licensed under Creative Commons Attribution 4.0 by the authors [66].

Therefore, this type of sensing method can be widely used for biomedical applications in constricted spaces. Fiber optic–based sensors allow easy sensor installation. In one study, TFBG was used to detect the variation in protein in the urine of rats [65]. Results were obtained using a coated TFBG embedded inside a microfluidic channel. The experiment was able to distinguish different kinds of urine. Results demonstrated a clear relationship between protein outflow and changes in the refractive index of the urine. This approach showed improvements in the detection of proteins at low concentrations [65]. The TFBG SPR sensor has been used for the detection of glycoprotein. Zhang et al [66] coated 10° TFBG with a 50-nm gold film to stimulate SPR on a sensor surface as shown in Figure 4. The sensor was able to distinguish between nonglycoproteins and glycoproteins. The TFBG-based sensor was also used to detect S-adenosyl-l-homocysteine (AdoHcy), with concentrations of up to 1 nM detected [67].

### Hybrid Whispering Gallery Mode Sensors

WGM resonators have been used for different applications, especially for high-sensitivity and resolution sensors [40,50-55]. The WGM microstructure can be made in various types of shapes such as spheres, cylinders, and toroid [40,50-55]. The WGMs of the resonator can be observed by coupling light to the resonator. These electromagnetic waves circulate near the internal edge of the resonator [54]. Therefore, the resonances are generated by the total internal reflection of the confined light and when the optical path of the light is a multiple integer of the wavelength [55]. The WGM shift caused by an excitation method can be used to determine the change in the measured quantities [40,56]. The WGMs can be tuned by excitation sources such as uniaxial stress and electric field. The theory is that the WGMs propagate across the pole of the spheres. Then, the deformation along the ends of the sphere and index of refraction change modify the position of WGM resonances [56,57]. WGM resonators have been developed using fused silica. Fused silica resonators have a quality factor (Q) of 10^9^ [58]. Silica resonators have been used, but have low sensitivity because they exhibit high Young modulus and therefore high resistance to any deformation. Different materials such as polydimethylsiloxanes have been used to address the issue of low sensitivity [59]. The WGM resonances can be observed using different techniques. For example, a study used a charge-coupled device camera and a spectrometer to observe the resonances, and therefore when doping WGM resonators with a laser dye material, the hybrid functions as tiny lasers that emit light under proper excitation conditions [40,50-55,67]. The light emitted from the resonator could then be coupled into the spectrometer using an optical lens setup [40]. Another study discussed a novel fiber-taper coupling system that couples light into microresonators. It has been observed that tapered optical fibers promote high coupling efficiency to the resonators. The experiment was completed with a silica microresonator coupled to a tapered optical fiber [34]. In a similar study, a silica microsphere resonator was critically coupled to a fiber taper. The fiber taper is useful because it allows for simple focusing and alignment of the input beam but uses the resonator as a passive element, which limits the application due to the presence of optical cablings [35,50].

**Figure 5:**
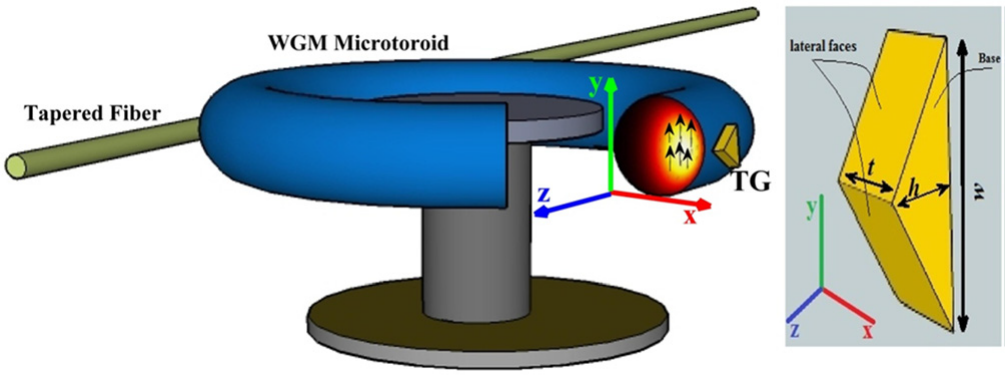
Geometrical scheme of whispering gallery mode (WGM) microtoroid with a gold triangular nanoprism bound to its surface. Reproduced from Nadgaran H, Afkhami Garaei M. Enhancement of a whispering gallery mode microtoroid resonator by plasmonic triangular gold nanoprism for label-free biosensor applications. Journal of Applied Physics 2015 Jul 28;118(4):043101. [doi:10.1063/1.4927266], with the permission of AIP Publishing [42].

Hybrid WGM methods have been analyzed in recent studies by coupling a WGM resonator to metal nanoparticles [40-42]. One study observed the effects of adding a gold nanoparticle to the equator of a microparticle. The motivation for this hybrid resonator was the need to rapidly detect pathogens. It works based on the principle of creating a plasmonic effect near the equator of the sensor, which enhances the already high-sensing capabilities of WGM-based sensors [41]. Other studies have used triangular gold nanoprisms coupled with WGM sensors. In one case, a gold triangular nanoprism was placed inside a microtoroid WGM resonator. It was shown that the tips of the nanoprism had regions of great plasmonic enhancement. This type of plasmonic enhancement permits the detection of larger protein molecules with high precision as shown in Figure 5 [42].

In other studies, polymeric WGM–based spherical resonators have been doped with metal nanoparticles to lower the energy required to activate the sensor [40]. In this case, the plasmonic effect enhanced light emission and lowered the energy threshold required for the structure to lase with higher temporal duration and more stable amplitude of the optical resonances, enabling multiplexed capabilities [40].

Electrically controlled graphene has also been applied to improve the performance of a hybrid silver–silica microdisk resonator. Most notably, the Q factor (energy stored and energy loss ratio) was improved and had a sensitivity higher than 1000 nm/RIU. Therefore, the hybrid sensor has a huge potential for use as a refractometer [60]. In recent years, there has been a growing interest in utilizing hybridization whispering gallery microstructures with the plasmonic effect. The motivation for this hybrid concept is that the single plasmonic sensing generates low Q factors (higher losses), whereas the presence of a resonant structure overcomes this limitation, thereby increasing the sensitivity of these hybrid sensors [61]. One example of the application of hybrid WGM biosensors is in the determination of proteins. More specifically, it was used to quantify the amount of bovine serum albumin that is absorbed by the gold nanoparticles [62], making the hybrid sensor a perfect candidate for combining plasmonic and high-sensitivity resonant microstructures. In addition, Huckabay et al [63] used WGM resonators to analyze a biomarker for ovarian cancer (CA-125) in a buffer.

### Some Other Relevant Examples of SPR/TFBG Applications in the Biomedical Field

**Figure 6:**
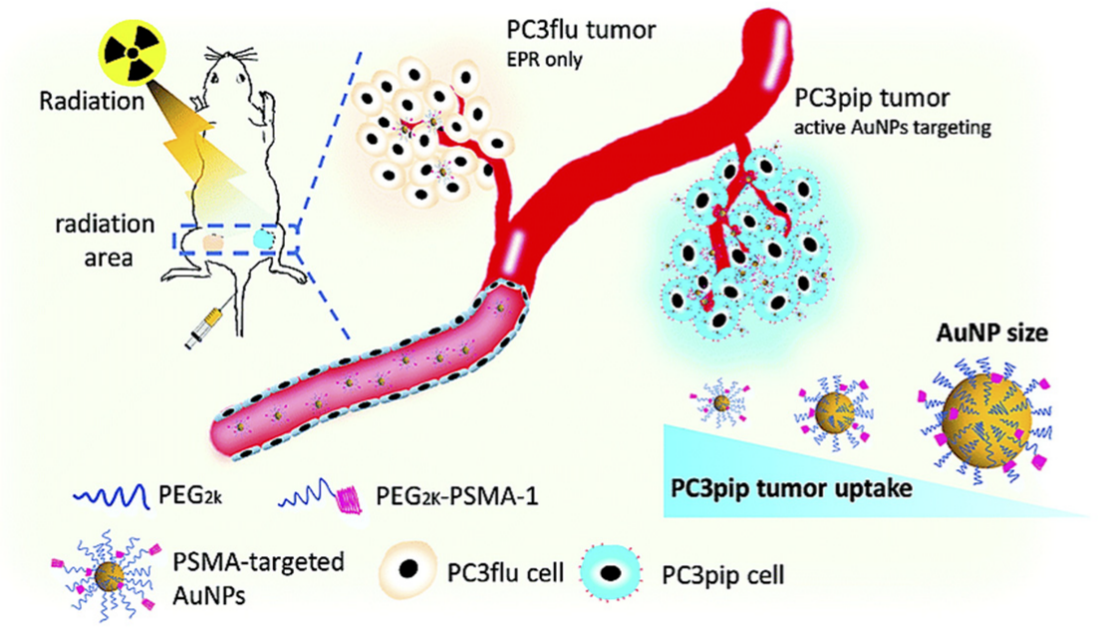
Schematic illustration of targeted prostate cancer radiotherapy using prostate-specific membrane antigen (PSMA)-targeted gold nanoparticles (AuNPs) of various sizes. With permission from the Royal Society of Chemistry, 2019 [69].

SPR is one of the prominent methods used for biomedical applications. Sharma [68] used a sensor based on SPR to detect the concentration of hemoglobin in human blood. Hemoglobin detection is an important medical procedure that has an impact on several clinical methods. Overall, this method of analyzing blood using SPR will lead to its use in blood analysis. Luo et al [69] used a plasmonic method employing gold nanoparticles, improving detection of tumor-targeted cells during X-ray radiotherapy (Figure 6).

Others have used gold nanorods to detect breast cancer biomarkers [5,70]. In addition to gold and silver, a few new plasmonic sensors based on different metals, such as magnesium, have been developed recently [71]. TFBG-based sensors have also been used to detect small biomarkers to diagnose lung cancers. The sensor was able to monitor the amplitude shift of sensitive spectrum modes of the TFBG SPR [72]. Furthermore, an immunosensor was used to detect biomarkers for risk stratification and prognosis of heart failure [73]. The detection of drugs and metabolites in patients currently remains a challenge and requires novel tools and methodologies. One study developed a diagnostic system based on silver nanoshells to detect metabolites in biofluids and identify whether patients had postoperative brain infection using embedded gold nanoparticles [74]. By contrast, ELISAs have been used to detect disease biomarkers at ultra-low concentrations. One study used this technique to detect HIV-2 capsid antigen p24 and prostate-specific antigen. This type of cost-effective technique can assist developing countries that require better methods to detect HIV infections. Therefore, it was noted that the plasmonic ELISA is a versatile method of detection for application in biomedical fields [19,75]. Silver nanocubes have also been applied for detecting lung cancer biomarkers, such as microRNAs. microRNAs, which are known to act as tumor suppressors, can be used for biomedical diagnosis. Zhang et al [49] developed a plasmonic nanoprobe technique to rapidly detect miR-21 biomarkers. miR-21 was used as a biomarker for diagnosing lung cancer early. The technique was based on the LSRP spectral shift that was caused by a change in the refractive index. Plasmon-based sensors are very versatile and in the near future it will be possible to see robust and cheap point-of-care devices for various daily monitoring and diagnosis of different medical conditions [19].

### Conclusions and Future Prospects

In this brief review paper, different plasmonic sensing methods and biosensing applications were discussed. Overall, biosensing is an attractive research area and novel sensing methods are being developed rapidly. Biosensing is a very powerful technique and will have a substantial impact on the biomedical community. This review summarized current methods and results that have influenced applications based on plasmonic biosensors. It was observed that the SPR is a notable principle for biosensing. This method is used for different applications such as for detection of sweat loss, biomarkers, and even hemoglobin concentration in human blood. Plasmon-based biosensors are versatile and will continue to be investigated and developed with technological advancements in the future to improve selectivity and robustness.

## Abbreviations

AdoHcy: S-adenosyl-l-homocysteine
ATR: attenuated total reflection
LSPR: localized surface plasmon resonance
SPR: surface plasmon resonance
TFBG: tilted fiber Bragg grating
WGM: whispering gallery mode
